# Advantages of Whole Genome Sequencing in Mitigating the *Helicobacter pylori* Antimicrobial Resistance Problem

**DOI:** 10.3390/microorganisms11051239

**Published:** 2023-05-08

**Authors:** Kartika Afrida Fauzia, Ricky Indra Alfaray, Yoshio Yamaoka

**Affiliations:** 1 Department of Environmental and Preventive Medicine, Oita University Faculty of Medicine, Yufu 879-5593, Japan; kartikafauzia@gmail.com (K.A.F.); rickyindraalfaray@gmail.com (R.I.A.); 2 Department of Public Health and Preventive Medicine, Faculty of Medicine, Universitas Airlangga, Surabaya 60115, Indonesia; 3 *Helicobacter pylori* and Microbiota Study Group, Institute of Tropical Disease, Universitas Airlangga, Surabaya 60115, Indonesia; 4 Division of Gastroentero-Hepatology, Department of Internal Medicine, Faculty of Medicine-Dr. Soetomo Teaching Hospital, Universitas Airlangga, Surabaya 60115, Indonesia; 5 Department of Medicine, Gastroenterology and Hepatology Section, Baylor College of Medicine, Houston, TX 77030, USA; 6 Borneo Medical and Health Research Centre, University Malaysia Sabah, Kota Kinabalu, Sabah 88400, Malaysia; 7 Research Center for Global and Local Infectious Diseases, Oita University, Yufu 879-5593, Japan

**Keywords:** *Helicobacter pylori*, whole genome sequencing, antimicrobial resistance, infectious disease

## Abstract

*Helicobacter pylori* antimicrobial resistance is a critical public health issue. Typically, antimicrobial resistance epidemiology reports include only the antimicrobial susceptibility test results for *H. pylori*. However, this phenotypic approach is less capable of answering queries related to resistance mechanisms and specific mutations found in particular global regions. Whole genome sequencing can help address these two questions while still offering quality control and is routinely validated against AST standards. A comprehensive understanding of the mechanisms of resistance should improve *H. pylori* eradication efforts and prevent gastric cancer.

## 1. Introduction

The World Health Organization (WHO) has designated the global rise of antimicrobial resistance (AMR) and the lack of adequate alternative therapies a global public health emergency [1]. AMR has the greatest significance in settings with limited resources, which are typically low- and middle-income countries that often have poor access to alternative medicines and, likely, a higher prevalence of multidrug-resistant bacterial strains [2]. This situation is applicable to *Helicobacter pylori*, where a high prevalence of antimicrobial resistance is often observed in such countries [3]. *H. pylori* has been listed by WHO as one of the 20 pathogens that pose the greatest threat to human health because of its drug resistance [4]. *H. pylori* causes gastrointestinal disorders, such as duodenal ulcers and gastric cancer, and infection by this bacterium is widely underestimated [5,6]. Insufficient epidemiological surveillance of *H. pylori* infections and antimicrobial resistance exists. The Maastricht consensus suggests that antibiotic use in an area must be adjusted to the pattern of antimicrobial resistance [7].

This editorial review includes articles published in the Special Issue of Microorganisms on Antimicrobial Resistance in *Helicobacter pylori*. Molecular epidemiology reports on *H. pylori* infection are discussed in detail. The key aspect of these reports is the use of whole genome sequencing (WGS) to further evaluate the mechanisms of antimicrobial resistance and the use of traditional antimicrobial susceptibility tests. This type of epidemiological report is essential in defining an appropriate therapeutic strategy to eradicate *H. pylori*.

## 2. Advantages of Whole Genome Sequencing

Phenotypic antimicrobial susceptibility testing (AST) determines whether bacterial isolates cultured in vitro grow in the presence of a specific antimicrobial agent at a specified concentration. Agar dilution and E-test experiments provide the minimal inhibitory concentration of an antibiotic that inhibits the growth of tested bacterial isolates. AST results provide crucial clinical care and surveillance information, but no direct information regarding resistance mechanisms, transmission routes, or pathogen evolution.

Next generation sequencing (NGS) technology is a fast and cost-effective approach that covers whole genome prediction and infectious disease surveillance of bacteria [8]. WHO has used WGS as the primary surveillance tool in the GLASS (Global Antimicrobial Resistance and Use Surveillance System) project to characterize pathogens and their mechanism of resistance and transmission [2]. Nonetheless, WGS cannot be used as a substitute for phenotypic methods in determining resistance. This approach aims to improve the global understanding of antimicrobial resistance patterns and underpin the development of effective interventions. Therefore, combining WGS with phenotypic methods provides a more comprehensive approach to antimicrobial resistance surveillance.

There are at least two main benefits of using WGS for monitoring antimicrobial resistance:

(1) Detect the geographical distribution of AMR and identify regions that have the potential to spread resistance.

AMR originates and spreads over measurable timescales. Monitoring the population structure of recognized infections enables a focused response to developing high-risk clones. A high-risk clone is a genetically homogeneous population of a bacterium with the same crucial resistance mutations and genes, rendering them resistant to one or more standard therapies because of their common lineage [9].

*H. pylori* has the highest recombination rate and can chronically infect the host [10]. This adaptation enables the characterization of the genome according to geographical location. Therefore, policies for treating *H. pylori* cannot be generalized worldwide. The surveillance of AMR infections in each country is essential for making informed policy decisions and implementing effective public health measures to combat *H. pylori* AMR. Adding molecular surveillance employing WGS to phenotypic surveillance of AMR can be beneficial. Implementing efficient infection control methods should enable a thorough understanding of the epidemiology and causes of AMR. Data provided to worldwide AMR surveillance initiatives enhance the coverage on a global scale. WGS of *H. pylori* is being constructed in various areas worldwide; thus, the distribution of the antibiotic resistome can be mapped from publicly available genomes [11].

(2) WGS enables the identification of the mechanism of resistance.

WGS identifies novel genetic determinants that can be targeted for new antimicrobial discoveries [12]. WGS-based methods comprehensively identify point mutation, gene rearrangement and other variants that can be varied among clinical isolates. Various antibiotics target specific proteins in bacteria [13]. Chromosomal mutations or acquisition of genetic material from the environment, such as a plasmid, phage, and/or transposon, can initiate resistance [11,14]. Mechanisms associated with gaining resistance include gene alterations that encode antibiotic targets, an increase in efflux pump activity and a decrease in cell permeability [14]. The WGS approach can evaluate multiple genes simultaneously.

## 3. Epidemiology Findings

**Figure 1:**
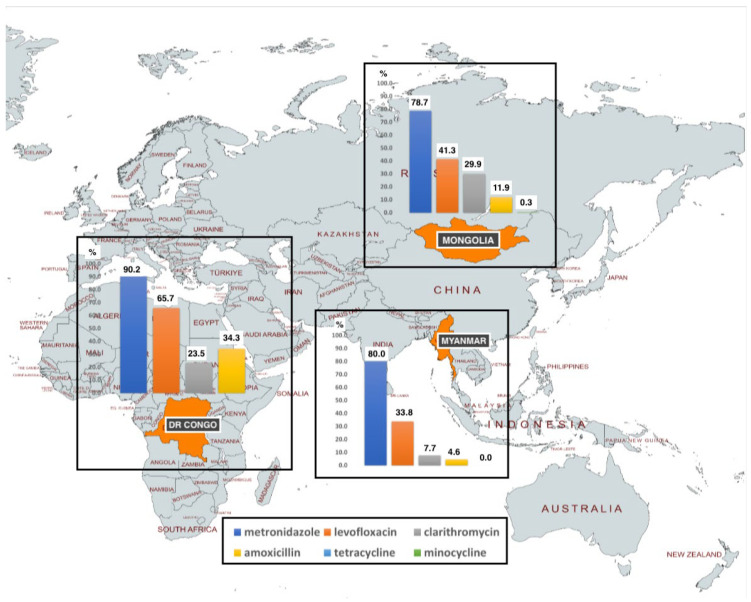
The map of antimicrobial resistance prevalence in Myanmar, Mongolia, and DR. Congo. The highest prevalence of resistance that are observed in all countries were metronidazole and followed by levofloxacin.

Articles published by Kabamba et al., Azzaya et al. and Subsomwong et al. present data from the Democratic Republic (DR) of Congo, Mongolia, and Myanmar, respectively (Figure 1) [15,16,17]. These studies provide the latest antimicrobial resistance prevalence data, which provide guidelines for treating H. pylori in each country.

Kabamba et al., Azzaya et al., and Phawinee et al. also assessed phenotypic AMR and its genetic determinant. A WGS-based approach was used to predict the phenotypic AST to amoxicillin (AMX), clarithromycin (CLA) and levofloxacin (LEVO). The practicality of the WGS approached to identify resistance were also evaluated. To find plausible links with resistance to each assessed drug, individual mutations from clinical isolates were compiled from 102 H. pylori isolates from Kinshasa, DR Congo, of which, 49.5% were successfully isolated and evaluated for phenotypic AST. 73.5% of the strains were resistant to multiple antimicrobials. 96.1% of strains were resistant to at least one antimicrobial. The resistance prevalence MTZ, LEVO, CLA, and AMX in DR. Congo were 90.2% 65.7%, 23.5%, 34.3%, respectively.

Moreover, using the agar dilution test, Azzaya et al. reported resistance toward metronidazole (MTZ), LEVO, CLA, AMX, and minocycline. The resistance prevalence was 78.7%, 41.3%, 29.9%, 11.9%, and 0.28%, respectively (Figure 1). An amount of 51.3% of the studied isolates exhibited multidrug resistance. Subsomwoong et al. evaluated the susceptibility of H. pylori to MTZ, LEVO, CLA, AMX, and tetracycline in Myanmar. Using the E-test, the resistance rates were 80%, 33.8%, 7.7%, 4.5%, and 0%, respectively (Figure 1).

In general, CLA resistance is increasing, which requires the design of an alternative regimen. Additionally, MTZ resistance is also extremely high, and levofloxacin resistance is relatively high. The high rate of multidrug resistance highlights the urgent need for effective antibiotic stewardship programs and alternative treatment strategies against H. pylori infections in these countries. Further studies are warranted to investigate the underlying resistance mechanisms, which should facilitate the development of new therapeutic options.

## 4. Identification of Well-Known and Novel Resistances

### 4.1. Amoxicillin Resistance

Studies observed that phenotypic AMX resistance is mainly related to mutations that alter the protein encoded by the penicillin-binding protein *pbp1A* gene [18] Mutations in *pbp1a* have been found in three countries. Interestingly, the novel mutations found in each country are diverse.

In silico docking analysis revealed that residues involved in binding AMX in PBP1a are Gly367, Ala369, Ile370, Lys371, Tyr416, Ser433, Thr541, Thr556, Gly557, Thr558, and Asn560 [18]. The mutations found in Myanmar are mainly located adjacent to this binding site. Mutations in locus 414 are found in AMX resistance in all countries and are adjacent to the motif SXN433-5 [19]. Adjacent to motif SXN433-5, novel mutations D465K and V471M were found in Myanmar; whereas A474T were found in DR Congo. The KTG555-7 motif was reportedly involved in forming and stabilizing an active cleft of the penicillin-binding protein [19]. The mutations in these adjacent regions, i.e., N562Y and T556S, were found in three countries. N562H and T558S are novel mutations found in DR Congo. Moreover, close to the SXXK368-71 motif, the novel mutation V374L in PBP1A was observed in Mongolia.

### 4.2. Clarithromycin Resistance

Thus, *23S rRNA* is the main target of clarithromycin [20]. The mutation of alleles at codon positions 2142 to 2144 in the sequence encoding domain V of *23S rRNA* explains the resistance phenotype. This mutation almost perfectly agrees with the phenotypic CLA resistance found in DR Congo. A similar result was also observed in Mongolia. 68.4% of resistance strains in Mongolia contain the A2143G mutation. Novel mutations A1410G (strain Uvs 147), C1707T (strain UB 130), A2167G (strain Ke 136), and C2922T have been identified.

### 4.3. Levofloxacin Resistance

The inhibition target of LEVO is the quinolone-determining region (QRDR) of DNA gyrase (*gyrA* and *gyrB*) [13]. The well-known resistant-determinant mutations are located in loci 87 and 91 of *gyrA*. These mutations are most significant in Myanmar, Mongolia, and DR Congo. Interestingly, several putative mutations were found in DR Congo, such as A92T, A129T of *gyrA,* and R579C in *gyrB.*

### 4.4. Metronidazole Resistance

Multiple mutations related to resistance to MTZ have been identified in genes encoding putative MTZ-reducing enzymes (*rdxA* and *frxA*), DNA recombination and repair (*recA*) proteins and superoxide dismutase (*sodB*) in *H. pylori*. In the clinical isolates from DR. Congo, structural analysis revealed variable sequence deletions in the RdxA, leading to lower binding affinity for Flavin-mononucleotide cofactors and MTZ activity.

The results indicate that WGS can be used to predict antibiotic susceptibility. The high resistance documented in these countries demonstrates the need for susceptibility-test-guided therapy. In addition to the phenotypic AST, the mutations identified by WGS can be used to develop an alternative diagnostic test.

## 5. Concern for Resistance Determination by WGS

Without additional testing, WGS cannot detect an AMR phenotype. WGS can only detect genotypes already known to be resistant or variants very similar to known resistance genotypes. For species with well-characterized genomes, in silico prediction of AMR phenotypes using WGS data yields consistent results and can be an efficient replacement for phenotypic testing [21,22]. However, for highly diverse genomic content, such as *H. pylori*, the concordant cannot reach more than 95%. Therefore, when predicting AMR, a comparison of multiple bioinformatic approaches reveals a high level of concordance [22,23,24,25].

Standardizing WGS approaches and pipelines currently used for detecting AMR-related determinants is required. External quality assurance (EQA) or performance testing of laboratory procedures is crucial for guaranteeing results generated by different laboratories are comparable, given the wide range of WGS technologies available and the variances in laboratory conditions [26]. As part of EQA, labs often analyze the same group of well-characterized, widely used bacterial strains. Therefore, validation studies are necessary, especially when various methods are used.

Various tools are currently available for constructing complete bacterial genomes. The *H. pylori* genome consists of chromosomes and extrachromosomal segments, such as phages and plasmids, and is approximately 1.6 Mbp in length. In general, sequencing can be accomplished through short- or long-read approaches, or both. Illumina and MGI Platforms are the most prominent short-read sequencing platforms. PacBio and Oxford Nanopore are typically used for long reads [27]. The benefits of short-read sequencing are cost-effectiveness, precision, and support by a multitude of analytical tools and pipelines [28].

Nevertheless, natural nucleic acid polymers span eight orders of magnitude in length, and sequencing them in small, amplified segments makes it challenging to reconstruct and count the original molecules. Long-read sequencing can generate reads between 5000 and 30,000 bp in length and circumvent short reads during assembly. However, long-read sequencing has the disadvantage of a relatively high error rate and relatively higher sequencing costs [27]. Combining short- and long-read sequencing is one method for producing optimal sequences of whole genomes.

## 6. Validation of WGS Methods

In the study by Tshibangu-Kabamba et al., four distinct WGS-based methods applicable to detecting AMR-related genetic determinants were compared. Short reads allow more accurate analysis of variants, and several workflows can be used. A validation process is needed by comparing various workflows to achieve the best concordance to the draft genome constructed based on short and long reads.

To validate the WGS approaches used for identifying AMR genetic determinants, Tshibangu-Kabamba et al. compared how well four different processes work with high-throughput NGS data. They examined the output from Illumina Miseq that went through the assembly process or directly mapped to the references (read mapping). The results were compared with complete WGS of the same isolate obtained by combining PacBio and Illumina Hiseq long reads of one of genome from DR. Congo, DRC64. The genes and variants in putative AMR-related genes were evaluated. Generally, the methods compared are de novo assembly based and read mapping-based to the reference genome. The result showed that full-length genes extracted from the de novo assembled and annotated WGS displayed the highest degree of congruence with gold standard sequences of all AMR-related genes tested (Kappa = 1.0; T.P. and T.N. rates of 100%; *p* = 0.0001).

The above results demonstrate that the bioinformatics technique influences using short-read sequencing to find antimicrobial resistance mutations. Many databases, such as Resfinder, RGI, and AMRFinder, have been created to discover well-known antibiotic resistance [29,30]. Nevertheless, these databases do not cover all *H. pylori* mutations. Moreover, the genomes must all undergo the assembly process, which is performed independently. The Ariba pipeline, which leverages input from FASTQ scans to identify new and well-known variants in target genes, can also be used to detect changes related to antimicrobial resistance [31].

## 7. Conclusions

Antimicrobial resistance is a pervasive issue that requires diverse solutions. We must determine the prevalence of resistance by combining phenotypic methods with WGS. This combined approach is more illuminating than a phenotype test alone because it also affords information about the resistance mechanism and geographical distribution. All of these methods should be carried out to eradicate *H. pylori* and, thus, prevent gastric cancer.

## Data Availability

Not applicable.
